# Aging‐Induced Episodic‐Like Memory Impairment Could be Alleviated by Melatonin Treatment via Preserving Blood–Brain Barrier Integrity and Upregulating CRTC1

**DOI:** 10.1111/cns.70412

**Published:** 2025-04-25

**Authors:** Yanping Wang, Xinyu Zhang, Hui Guo, Shuxia Qian, Hailun Fang, Xiaoqiang Wu, Yufei Shen, Congying Xu, Beiqun Zhou, Chun Guo, Xudong Lu, Xiaoling Zhang, Xinchun Jin

**Affiliations:** ^1^ Department of Neurology The Second Affiliated Hospital of Jiaxing University Jiaxing China; ^2^ Institute of Neuroscience The Second Affiliated Hospital of Soochow University Suzhou China; ^3^ Department of Histology and Embryology, School of Basic Medical Sciences, Advanced Innovation Center for Human Brain Protection Capital Medical University Beijing People's Republic of China; ^4^ Department of Neurology Bengbu Medical College Bengbu China; ^5^ School of Biosciences University of Sheffield Sheffield UK

**Keywords:** aged mice, CRTC1, episodic‐like memory, melatonin, novel object recognition

## Abstract

**Background:**

Aging is accompanied by impairments in stimulus recognition, and decreased melatonin levels have been shown in aged mice and humans. These age‐related changes are associated with an increased risk of neurological diseases. In the present study, our aim is to investigate whether melatonin supplementation could ameliorate age‐related cognitive decline in aged mice.

**Methods:**

Mice were treated with melatonin or saline. The novel object recognition (NOR) task was used to provide a simultaneous assessment of object and object location memory, which is a component of episodic‐like memory. Blood–brain barrier (BBB) leakage was assessed using an Immunoglobulin G (IgG) leakage assay. Immunofluorescence and Western blot analyses were employed to investigate changes in protein levels.

**Results:**

We demonstrate that aging impairs memory in the NOR task, with concomitant decreases in the levels of synaptophysin (SYP), CREB‐regulated transcription coactivator 1 (CRTC1), and phosphorylated AMP‐activated protein kinase (p‐AMPK) levels within the prefrontal cortex (PFC) and hippocampus. Moreover, alongside compromised BBB integrity, aging results in the degradation of occludin in both the PFC and hippocampus. Our findings demonstrate that aging impairs memory performance in the NOR task, accompanied by reductions in SYP, CRTC1, and p‐AMPK levels within the PFC and hippocampus. Furthermore, alongside compromised BBB integrity, aging results in the degradation of occludin in both the PFC and hippocampus. More importantly, PDZ and LIM domain 5 (Pldim5) was upregulated in melatonin‐treated mice, and aging‐related memory impairment in the NOR task was significantly reduced in Pdlim5^−/−^ mice. Notably, 1 week of melatonin (10 mg/kg) treatment significantly improved memory, along with enhanced BBB integrity, Pdlim5 downregulation, and CRTC1 and p‐AMPK upregulation.

**Conclusions:**

Taken together, our findings suggest that melatonin ameliorates aging‐related memory decline in the NOR task by downregulating Pdlim5, maintaining BBB integrity, and upregulating CRTC1 and p‐AMPK in aged mice.

## Graphical abstract

Aging impairs memory in NOR by downregulating the expression of SYP, CRTC1, AMPK, and Pdlim5‐mediated BBB damage. Melatonin treatment improves memory by upregulating CRTC1 and AMPK, as well as maintaining the integrity of BBB by downregulating Pdlim5.

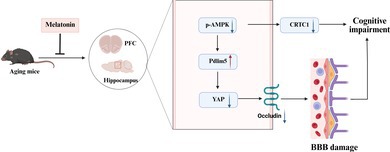

**Abbreviations**
AFMK: N1‐acetyl‐N2‐formyl‐5‐methoxykynuramine
AMK: N1‐acetyl‐5‐methoxykynuramine
AMPK: AMP‐activated protein kinase
BBB: blood–brain barrier
CRTC1: CREB‐regulated transcription coactivator 1
IgG: Immunoglobulin G
i.p.: intraperitoneally
LPS: lipopolysaccharide
NLR: novel object location recognition
NOR: novel object recognition
p‐AMPK: phosphorylated AMP‐activated protein kinase
Pdlim5: PDZ and LIM domain 5
PFC: prefrontal cortex
ROS: reactive oxygen species
SYP: synaptophysin
TJPs: tight junction proteins
YAP: yes‐associated protein

## 1 Introduction

Episodic‐like memory is context‐ and content‐specific and is sensitive to the accumulation of both Alzheimer‐like pathology and the effects of normal aging in mice [1] and humans [2]. Aging is accompanied by declines in stimulus recognition [3]. Specifically, older adults often exhibit impairments in recognition memory, which enables the retrieval of previously presented items [4] and may be required for familiarity and judgment recollection.

CREB‐regulated transcription coactivator 1 (CRTC1) is a potent modulator of cAMP response element (CRE)‐driven gene transcription [5], playing a crucial role in regulating longevity under conditions of limited energy. Aging‐induced dysregulation of CRCT1 is regarded as a probable target for slowing down aging‐associated memory loss [6]. In addition, aging has been shown to affect the activation of adenosine monophosphate‐activated protein kinase (AMPK) [7], a serine/threonine protein kinase that regulates cellular and organismal metabolism [8]. Of note, constitutive AMPK activity directly inactivates CRTC1 via phosphorylation, a crucial step in AMPK‐mediated longevity in *C. elegans* [9, 10].

The aging process disrupts the blood–brain barrier (BBB), causing both structural and functional damage, and BBB damage by extrinsic or intrinsic stimuli can lead to neurological diseases [11], and the BBB could be a target to reduce neurological disease [12]. We have recently shown that Pdlim5, an adaptor protein containing PDZ and LIM domain 5, is critically required for regulating lipid metabolism heterogeneity in Alzheimer disease [13], is functionally important in BBB damage after acute ischemic stroke [14], and Pdlim5/YAP critically mediates BBB disruption in acute ischemic stroke [15]. Of note, we have shown that melatonin supplementation exerts a protective effect against lipopolysaccharide‐induced BBB disruption in aged mice [16] and that melatonin treatment ameliorates ischemic stroke‐induced BBB damage [17]. Notably, melatonin and its metabolites can eliminate a variety of free radicals and exert beneficial effects on aging‐induced memory impairment [18]. For example, N1‐acetyl‐5‐methoxykynuramine, one of the melatonin metabolites, is known to improve long‐term object memory in aging mice [19]. Furthermore, melatonin treatment can attenuate memory deficits, neuroinflammation, and neurodegeneration produced by D‐galactose in aging mice models [18] and improve D‐galactose‐produced aging effects in mice [20].

Novel object recognition (NOR) is sensitive to the normal aging process in mice [1], and the NOR task has been employed to evaluate the different components of episodic‐like memory in mice [21] and rats [22]. In the current study, we aimed to examine whether melatonin supplementation can improve aging‐induced NOR memory loss and underpinning molecular mechanisms.

## 2 Materials and Methods

### 2.1 Animal

The animal procedures abided by animal care guidelines that are authorized by the University Committee on Animal Care of Soochow University (No. IACUC‐201611A355) and guidelines of the National Institutes of Health for the Care and Use of Laboratory Animals. The experimental mice consisted of young (9‐week‐old) and old (12 to 14‐month‐old) male C57BL/6J mice. Young mice were obtained from the Shanghai Laboratory Animal Center (SLAC), while old mice were acquired from the Soochow University Animal Experiment Center. During rearing, the number of mice per cage was maintained at 4–5 mice, the room temperature was maintained at 23°C ± 1°C, 12 h of alternating light/darkness, and the mice were assured of adequate and free access to food and water. There was no randomization or blinding of experimental treatments. Every attempt was made to reduce the pain and number of animals used according to the guidelines. The detail for the number of animals for every experiment was stated in relevant figure legends.

Pdlim 5^−/−^ mice, produced by focusing on the mouse ENH gene's third exon, have been described previously [23].

### 2.2 Drug Administration

A 2% ethanol with 0.9% saline was used as a solvent to prepare Melatonin (Sigma, St Louis, MO, USA) solution (Wang et al., 2017). Melatonin (10 mg/kg/day) or saline was given at 9:00–10:00 a.m. every 24 h via intraperitoneal injection for 1 week [24, 25]. Mice underwent training 24 h after the final administration.

### 2.3 Behavioral Training and Testing

#### 2.3.1 Novel Object Recognition (NOR)

A Plexiglas open‐field box (50 × 50 × 50 cm high) with white walls and a white floor was employed as the experimental apparatus. The mice's behavior was recorded by a video recorder. The stimuli that were presented to the mice were similar objects with different shapes, colors, and sizes. The mice were not able to remove the heavy objects.

There were three stages for the experiment: adaptation period, familiarity period, and test period [21]. Mice were adapted to the empty open‐field box for 2 days, with a 10‐min session per day during the adaptation period. In addition, 75% alcohol was used to clean the open‐field box and objects after each trial.

**FIGURE 1:**
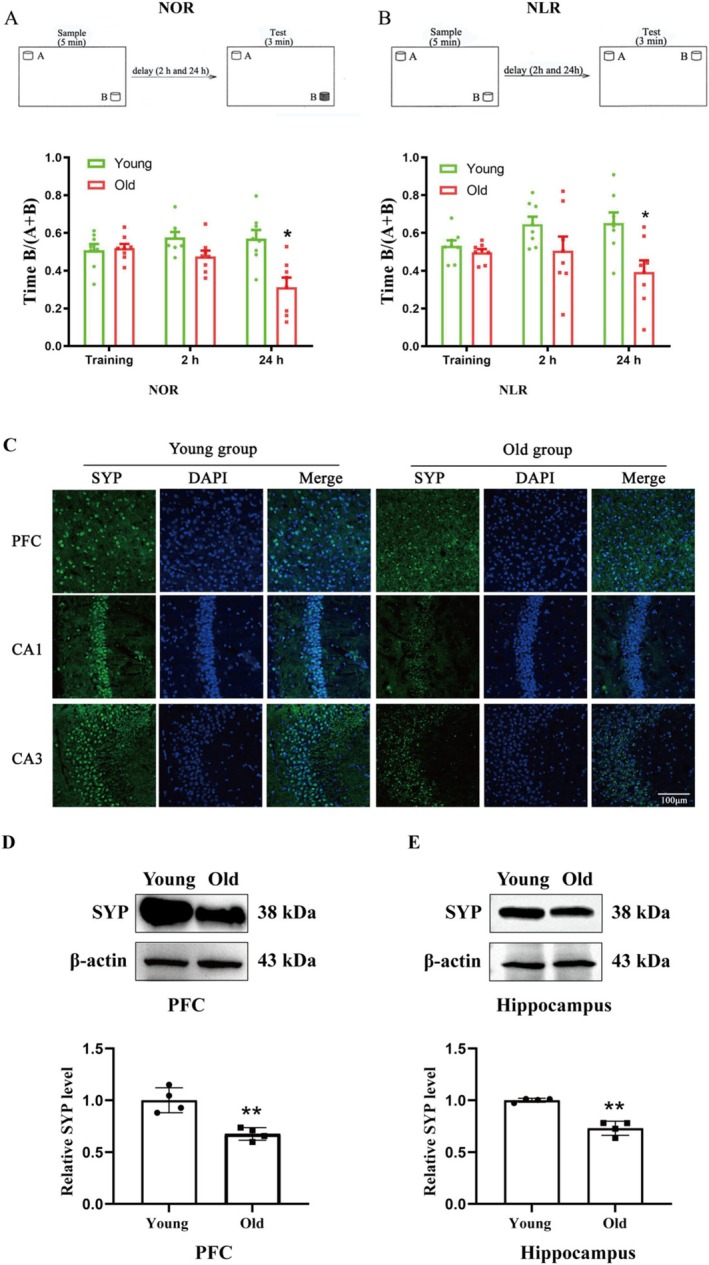
Effect of aging on recognition memory in NOR task and SYP expression. (A) Demonstration of sample and test of NOR memory. Time spent in novel object during training and 2 and 24 h test of NOR memory. (B) Demonstration of sample and test of NLR memory. Time spent in novel location during training and 2 and 24 h test of NLR memory. *N* = 8/group. (C) Representative fluorescence micrographs demonstrated SYP expression in the PFC and hippocampus. Aging significantly decreased SYP levels in the PFC and hippo. *N* = 3, scale bar = 100 μm. Representative western blot demonstrated the bands of SYP in PFC (D) (upper panel) and hippo (E). The relative band intensity of SYP was quantitated. Aging produced a significant decrease of SYP in the PFC (D, *p < 0.05 vs. young) and hippo (E, *p < 0.05 vs. young). *N* = 4/group. Data were expressed as mean ± SEM.

Following the sample phase (acquisition), preference tests were done at 2 and 24 h (Figure 1A). Two similar items were positioned at the opposing corners of the open‐field box during the sampling phase. After it was placed into the box facing the wall, after 5 min of exploration, the mouse was placed in the box and two objects were shown in the same location as during the sample phase for the 3‐minute test: one object was identical to the one used during the acquisition period, while the other was a novel object.

#### 2.3.2 Novel Object Location Recognition (NLR)

This task assessed the mouse's capacity to identify the changed position of a previously encountered object. The sample phase lasted 5 min, allowing the mouse to explore the object. The two corners of the open‐field box were used to position the objects (Figure 1B). The 3‐min test phase commenced 2 and 24 h after the sample phase. Two objects in the test phase were identical in shape, color, and size. One object was positioned identically to its placement in the sample phase, while the other object was placed in a new position.

### 2.4 Experimental Procedures

#### 2.4.1 Experiment 1

This experiment investigated the aging‐related memory changes in novel object recognition tasks and the expression of aging‐related proteins SYP, CRTC1, and AMPK.

#### 2.4.2 Experiment 2

This experiment investigated the aging‐related BBB damage, the integrity of blood microvessels, as well as the expression of occludin.

#### 2.4.3 Experiment 3

This experiment checked the aging‐induced expression of Pdlim5 and the function of Pdlim5 in aging‐induced memory impairment.

#### 2.4.4 Experiment 4

This experiment explored the impact of systemic melatonin administration (10 mg/kg) on NOR and NLR memory in 12‐month‐old mice following 7 days of treatment [16].

#### 2.4.5 Experiment 5

This experiment examined the impact of systemically administered melatonin on the expression of SYP, CRTC1, AMPK, occludin, YAP, and Pdlim5.

### 2.5 Assessment of BBB Permeability Using Immunoglobulin G (IgG) Extravasation Assay

IgG extravasation assay was conducted to assess BBB integrity as previously reported [17]. Briefly, 20‐μm‐thick sections were fixed with 4% paraformaldehyde for 20 min at room temperature. Subsequently, sections were incubated with Cy‐3‐conjugated Affinity Pure Goat Anti‐Mouse IgG (1:200 dilution, KPL, Gaithersburg, MD, USA) for 2 h. Following staining, sections were mounted with a glass coverslip and imaged using an LSM700 confocal microscope (Carl Zeiss).

**FIGURE 2:**
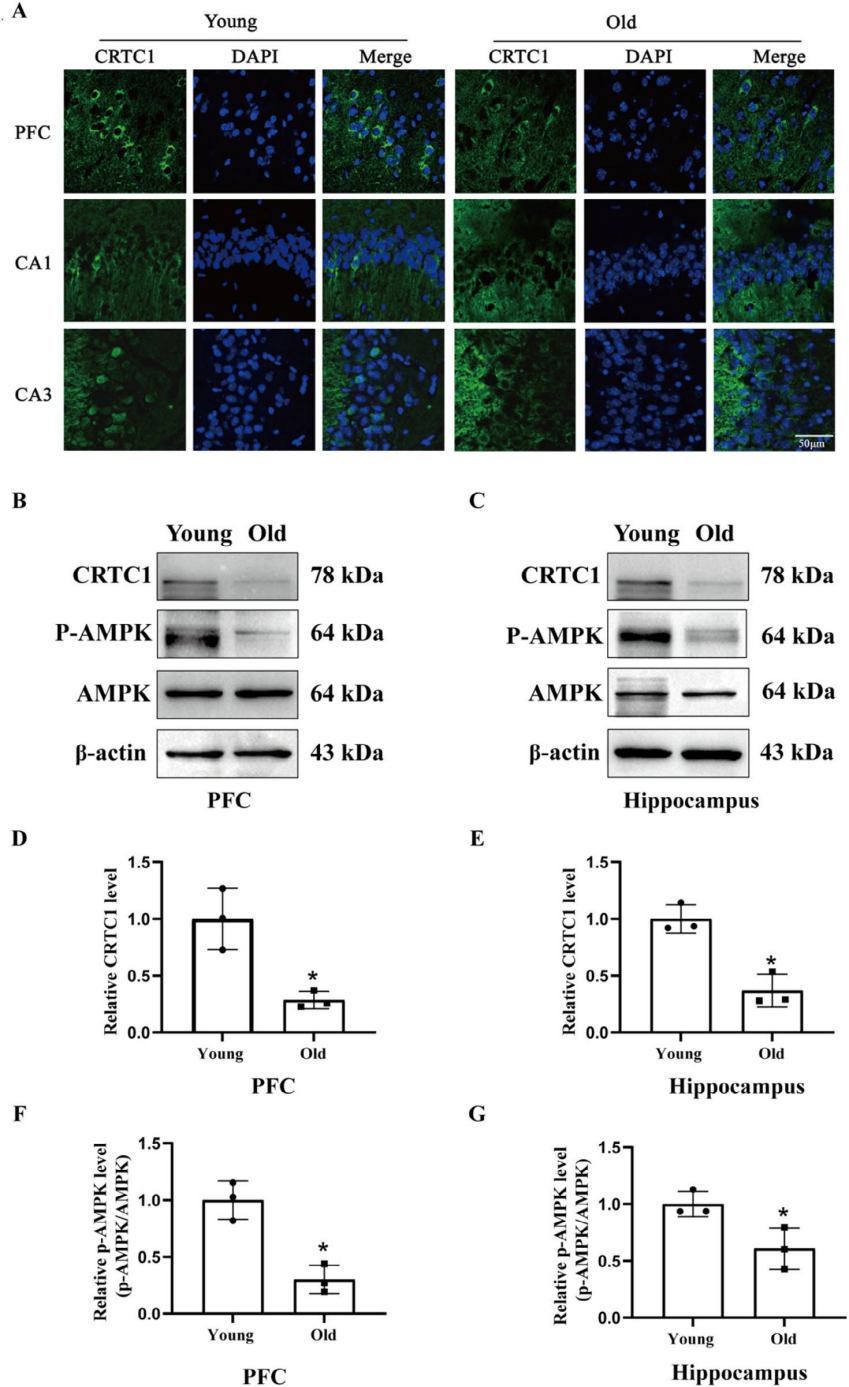
Effect of aging on the protein level of CRTC1, p‐AMPK, and AMPK. (A) Representative fluorescence micrographs showed CRTC1 distribution in the PFC and hippo. Aging significantly decreased the level of CRTC1 in the PFC and hippocampus. *N* = 3, scale bar = 50 μm. Representative western blot displayed the bands of CRTC1, p‐AMPK/AMPK in PFC (B) and hippo (C). The relative band intensity of CRTC1 and p‐AMPK/AMPK were quantitated. Aging significantly reduced the protein levels of CRTC1 (D) and p‐AMPK/AMPK (F) in PFC (*p < 0.05 vs. young), and the protein levels of CRTC1 (E) and p‐AMPK/AMPK (G) in the hippo (*p < 0.05 vs. young). *N* = 3/group. Data were shown as mean ± SEM.

### 2.6 Isolation of the Cerebral Microvessels

**FIGURE 3:**
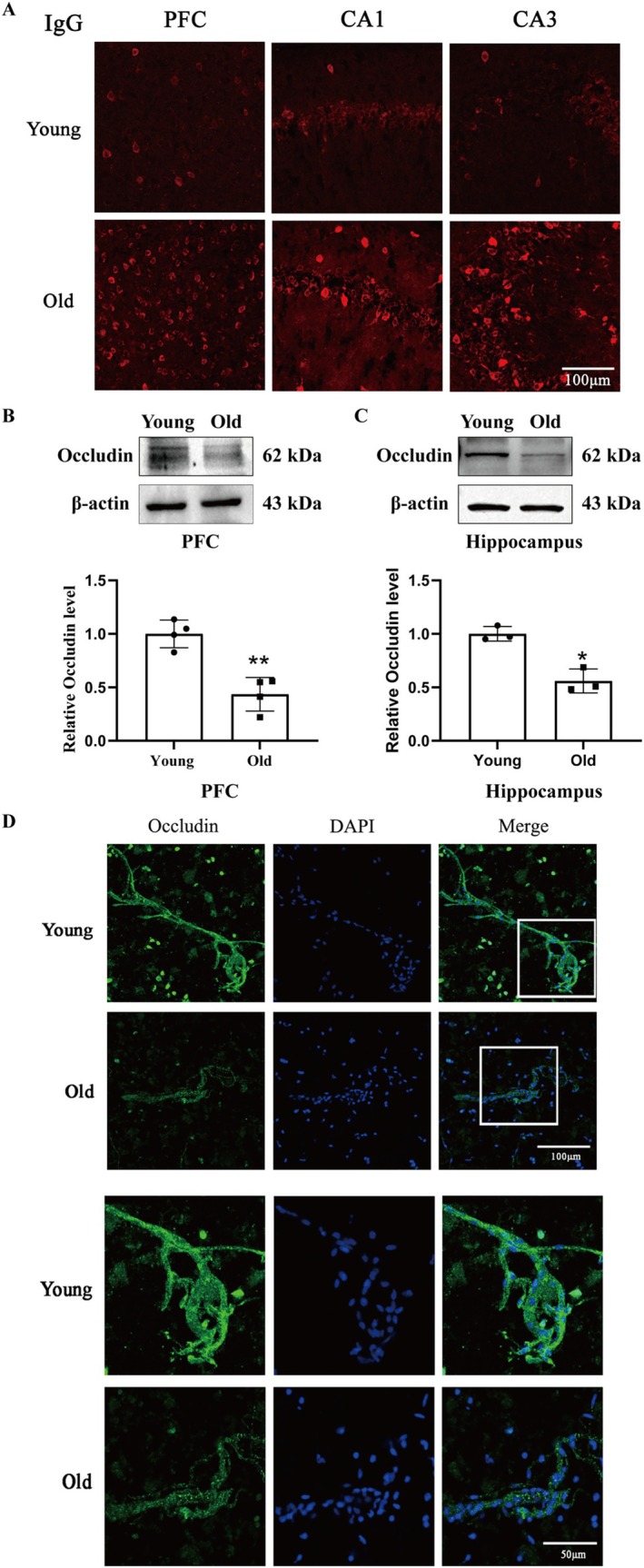
Effect of aging on BBB permeability, expression of occludin, and integrity of cerebral microvessel. (A) Representative fluorescence micrographs showed IgG leakage in the PFC and hippo of young and old mice. Significant IgG leakage was observed in old mice compared to young mice. Scale bar = 100 μm, *n* = 3/group. Representative immunoblot showed the bands of occludin in the PFC (B, upper panel) and hippocampus (C, upper panel). The relative band intensity of occludin was quantitated. Aging significantly reduced the protein levels of occludin in the PFC (B, lower panel, *p < 0.05) and the hippocampus (C, lower panel, *p < 0.05). *N* = 3–4/group. Data were shown as mean ± SEM. (D) Aging induced a significant impairment in the integrity of microvessels. Scale bar = 50 μm, *n* = 3/group.

Cerebral microvessel isolation was done as previously reported [26]. Briefly, half of the brain was dissected, meninges were removed, and cortical and subcortical tissues were collected. A homogenizer was applied to homogenize the tissue d in ice‐cold PBS. A 41 μm nylon mesh (Spectrum, Irving, TX, USA) was used to filter the homogenate to isolate microvessels. Following three PBS washes, microvessels were collected by centrifugation (4000 g, 10 min, 4°C). The microvessels were further purified by resuspending the pellet in 15% dextran T‐500, followed by the addition of 20% dextran T‐500. After centrifugation (25,000 g, 10 min, 4°C), the microvessel pellet was collected and stored at −80°C. The purity of the isolated microvessels was confirmed by immunostaining for occludin (1:100, Invitrogen, Carlsbad, CA, USA). Figure 3D shows a representative fluorescent micrograph of isolated microvessels stained with anti‐occludin antibody followed by FITC‐conjugated secondary antibody, as described previously [27].

### 2.7 Immunofluorescence Analysis for CRTC1, Occludin, and SYP

Immediately following memory testing, mice received a transcardial perfusion of PBS to remove blood, and then 4% PFA for tissue fixation. Brains were sectioned at 20 μm thickness using a cryostat. Sections were permeabilized with 0.25% Triton X‐100 in PBS after being fixed in 4% PFA and cleaned in PBS. A solution comprising 0.3% Triton X‐100, 1% BSA, and 5% goat serum was used to prevent nonspecific binding. Sections were incubated with primary antibodies for an entire night at 4°C with primary antibodies against SYP (1:200), CRTC1 (1:200), and occludin (1:100), all obtained from Invitrogen (Carlsbad, CA, USA). Subsequently, sections were treated for 2 h at room temperature with appropriate Fluor‐conjugated secondary antibodies (1:1000 dilution, Boster, Wuhan, China). Images were acquired using a Zeiss LSM 700 laser scanning confocal microscope.

### 2.8 Western Blot to Detect SYP, p‐AMPK, AMPK, CRTC1, Occludin, Pdlim5, and YAP

Immediately after the memory test, mice ice‐cold PBS was transcardially infused into the mice. Brain tissues, specifically from the prefrontal cortex (PFC) and hippocampus, were dissected and collected for subsequent protein expression analysis. RIPA buffer containing protease and phosphatase inhibitors (Beyotime) was used to mechanically homogenize the mice's brain tissues. The BCA was used to determine the protein content with high‐temperature boiling denaturation as per the manufacturer's instructions. Thirty microgram of total protein was resolved on a 12% SDS‐PAGE gel, transferred to a PVDF membrane (Millipore, Billerica, MA, USA), and blocked with 5% skimmed milk in TBS‐T. Membranes were then incubated overnight at 4°C with primary antibodies against p‐AMPK (1:1000, CST), AMPK (1:1000, CST), Pdlim5 (1:1000, Abcam), SYP (1:1000, CST), CRTC1 (1:1000, Abcam), occludin (1:500, Invitrogen), and YAP (1:500). After washing with TBS‐T, membranes were incubated with HRP‐conjugated secondary antibodies (anti‐rabbit or anti‐mouse, 1:2000, Boster) for 1 h at room temperature. The SuperSignal West Pico HRP Substrate Kit (Pierce, Rockford, IL, USA) was used in an ECL detection system to view protein bands. Relative density values for each band were measured using Image J.

### 2.9 Statistical Analysis

SPSS software version 17.0 was used for statistical analyses. All data are presented as mean ± SEM. One‐way or two‐way ANOVAs were employed for multiple comparisons, followed by Newman–Keuls post hoc tests to identify significant differences between groups. Statistical significance was determined at the *p* < 0.05 level.

## 3 Results

### 3.1 Effect of Aging on Recognition Memory in NOR Task and Synaptophysin Expression

It is well established that aging impairs episodic memory in humans [2] and mice [1]. Episodic memory, a crucial cognitive function, encompasses several key components. These include memory for novel object recognition (NOR), which pertains to recognizing and remembering specific objects or individuals, and memory for novel location recognition (NLR), which involves recalling the environment or context in which a particular experience happened.

Using NOR, we observed that aging significantly impaired the 24 h (*p* < 0.05, Figure 1A), but not 2 h (*p* > 0.05, Figure 1A) NOR memory, which was reflected in decreased exploration time for the less recently experienced object compared to the recently experienced object. Aging also significantly impaired 24 h NLR memory (*p* < 0.05, Figure 1B), which was reflected in decreased exploration time for objects in a novel place.

In addition, synaptophysin (SYP), which is related to synaptic plasticity within memory formation [27], was significantly decreased in PFC (Figure 1D) and hippo (Figure 1E) in aged mice compared to those in the young mice, as demonstrated by immunofluorescence (Figure 1C) and western blot (Figure 1D,E) analysis.

### 3.2 Effect of Aging on the Expression Level of Memory‐Associated Proteins CRTC1 and p‐AMPK

Aging plays a crucial role in life span extension. Studies have shown that aging significantly decreases the expressions of CRTC1 [9] and AMPK in *C. elegans* [7]. Our immunofluorescence results showed that aging led to significantly decreased CRTC1 levels in both PFC and hippo (Figure 2A). The western blot results verified a significant decrease in the level of CRTC1 (*p* < 0.05) and p‐AMPK (*p* < 0.05) in both PFC (Figure 2B,D,F) and hippocampus (Figure 2C,E,G), suggesting that CRTC1 and AMPK may play critical roles in aging‐induced memory impairment in the NOR task.

### 3.3 Effect of Aging on BBB Permeability, Occludin Expression, and the Integrity of Cerebral Microvessels

BBB dysfunction, as an early affair in the old human brain, initiates in the hippocampus and might play a role in cognitive deficit, including memory loss [28]. Here, we explored the impact of aging on the BBB integrity in mice. The immunofluorescence results showed that aging significantly impaired BBB integrity in the PFC and hippocampus, which was reflected by the increased IgG leakage in the PFC and hippocampus (Figure 3A). In addition, aging significantly decreased the level of the tight junction proteins (TJPs) occludin in the PFC (*p* < 0.05, Figure 3B) and hippocampus (*p* < 0.05, Figure 3C), suggesting that occludin degradation‐mediated impairment of BBB integrity may be critically involved in aging‐produced memory deficit in the NOR task.

We next checked the integrity of the microvessels by isolating cerebral microvessels. Immunofluorescence results showed that compared with the microvessels from the young mice, aging caused a significant impairment of the microvessels' integrity (Figure 3D).

### 3.4 Effect of Aging on YAP and Pdlim5 Expression in Hippocampus and PFC and Effect of Aging on Memory in the NOR Task in Pdlim5^−/−^ Mice

**FIGURE 4:**
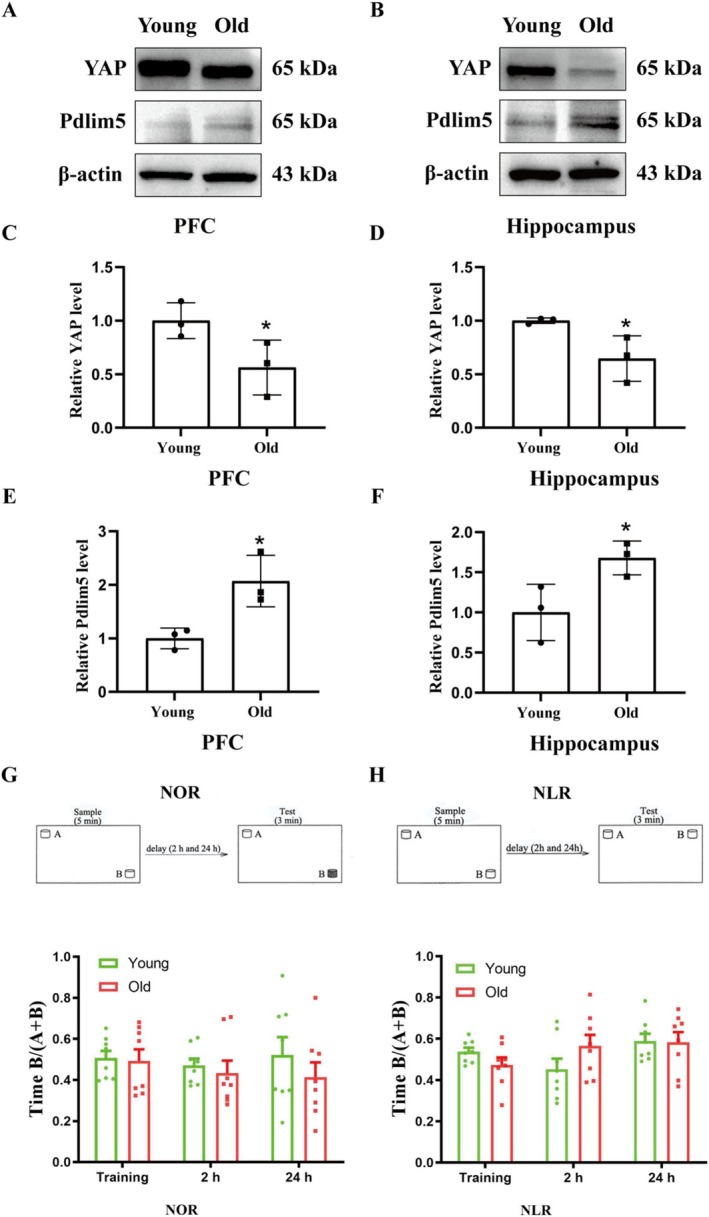
Effect of aging on the expression of YAP, Pdlim5, and NOR in young and old Pdlim5^−/−^ mice. Representative immunoblot exhibited the bands of YAP and Pdlim5 in PFC (A) and hippo (B). The relative band intensity of YAP and Pdlim5 were quantitated. Aging significantly reduced the protein levels of YAP in the PFC (C) and hippo (D) and upregulated the protein levels of Pdlim5 in PFC (E) and hippo (F). *p < 0.05 vs. young. *N* = 3/group. (G, H) Demonstration of sample and test of NOR memory (G, upper panel) and NLR memory (H, upper panel) in young and old Pdlim5^−/−^ mice. (E, F) Time spent in novel object during training and 2 and 24 h test of NOR memory (G, lower panel) and NLR memory (H, lower panel) in young and old Pdlim5^−/−^ mice. *N* = 8/group. Data were shown as mean ± SEM.

YAP mitigates BBB disruption following cerebral ischemia/reperfusion injury [29] and Pdlim5 is known to play a crucial role in BBB damage after acute ischemic stroke [14]. In addition, Pdlim5 has been shown to regulate YAP [30]. Here, we compared the expression of YAP and Pdlim5 in young versus aged mice. We demonstrated that aging resulted in significantly decreased YAP expression in the PFC (*p* < 0.05, Figure 4A,C) and hippocampus (*p* < 0.05, Figure 4B,D) and increased the expression of Pdlim5 in the PFC (*p* < 0.05, Figure 4A,E) and hippocampus (*p* < 0.05, Figure 4B,F).

Compared with the young Pdlim5^−/−^ mice, aging did not significantly impair the NOR (*p* > 0.05, Figure 4G) and NLR memory (*p* > 0.05, Figure 4H), indicating that Pdlim5 plays a crucial role in aging‐induced memory impairment in the NOR task.

### 3.5 Effect of 1‐Week Melatonin Treatment on Memory in NOR Task in 12‐Month‐Old Mice

**FIGURE 5:**
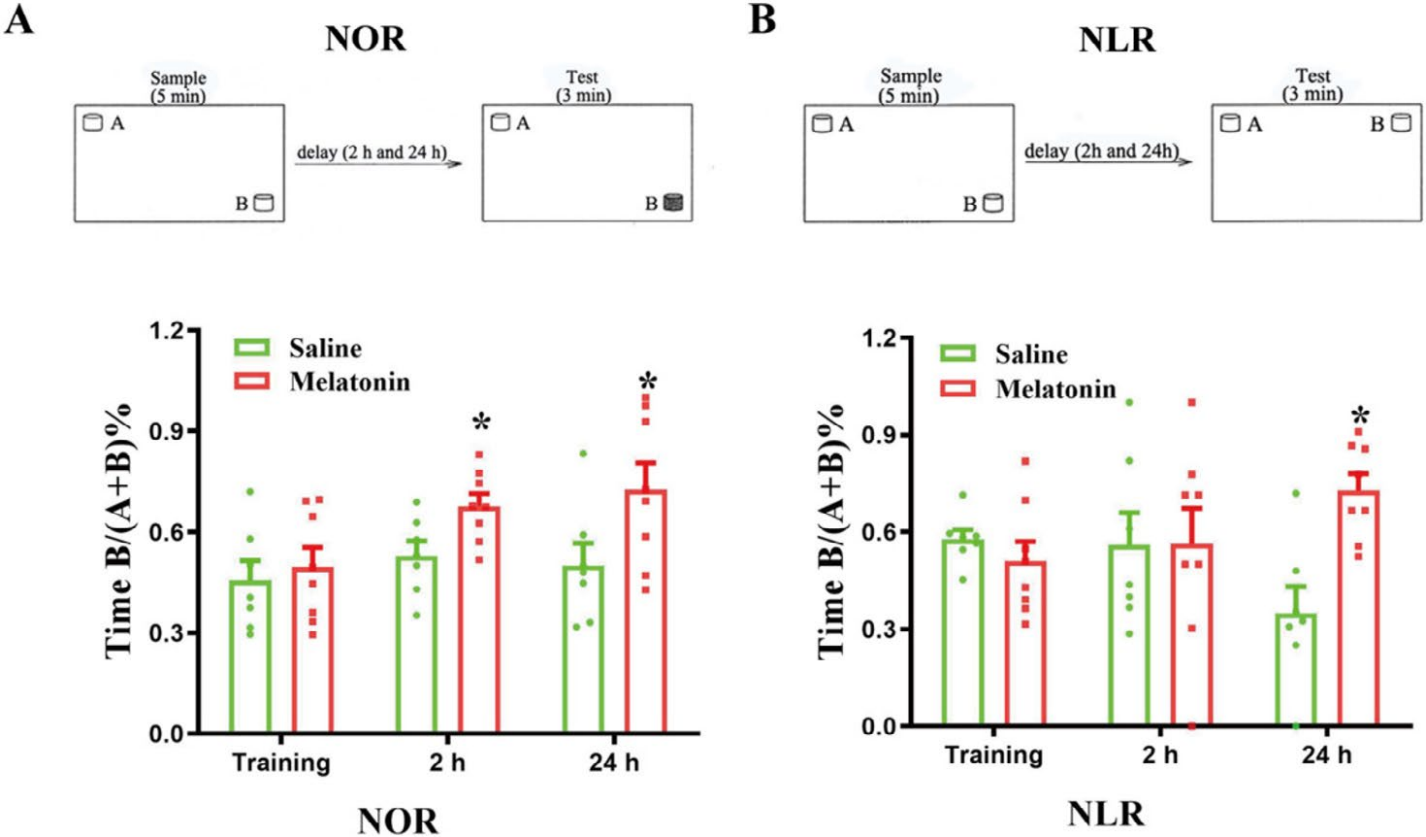
Melatonin improves NOR and NLR memory in the NOR task of 12‐month mice. (A) Time spent in novel object recognition (NOR) during training and 2 and 24 h test of NOR memory. One‐week melatonin treatment significantly improved 24‐h NOR memory of 12‐month mice in the NOR task. (B) Time spent in novel location recognition (NLR) during training and 2 h and 24 h test of NLR memory. One‐week melatonin treatment had significant improvement in 24‐h NLR memory of 12‐month mice in the NOR task. *N* = 8/group. Data were shown as mean ± SEM.

Aging is accompanied by a significant decline in melatonin in rats [31] and humans [32]. Of note, melatonin is reported to improve spatial and episodic‐like memory in the Barnes maze and NOR tasks [33]. Melatonin treatment (10 mg/kg) for 1 week has been demonstrated to mitigate LPS‐produced BBB damage in aged mice [16]. Here, we checked whether supplementation with 1‐week melatonin (10 mg/kg/day) could improve memory performance in the NOR task. As shown in Figure 5, 1‐week melatonin treatment significantly improved 24‐h NOR memory (*p* < 0.05, Figure 5A) and NLR memory (*p* < 0.05, Figure 5B) in the NOR task. It is of note that 1‐week melatonin treatment affects short‐term (2‐h) NOR memory but not NLR memory (*p* > 0.05, Figure 5A,B).

### 3.6 Effect of 1‐Week Melatonin Treatment on the Expression Level of SYP, CRTC1, and p‐AMPK

**FIGURE 6:**
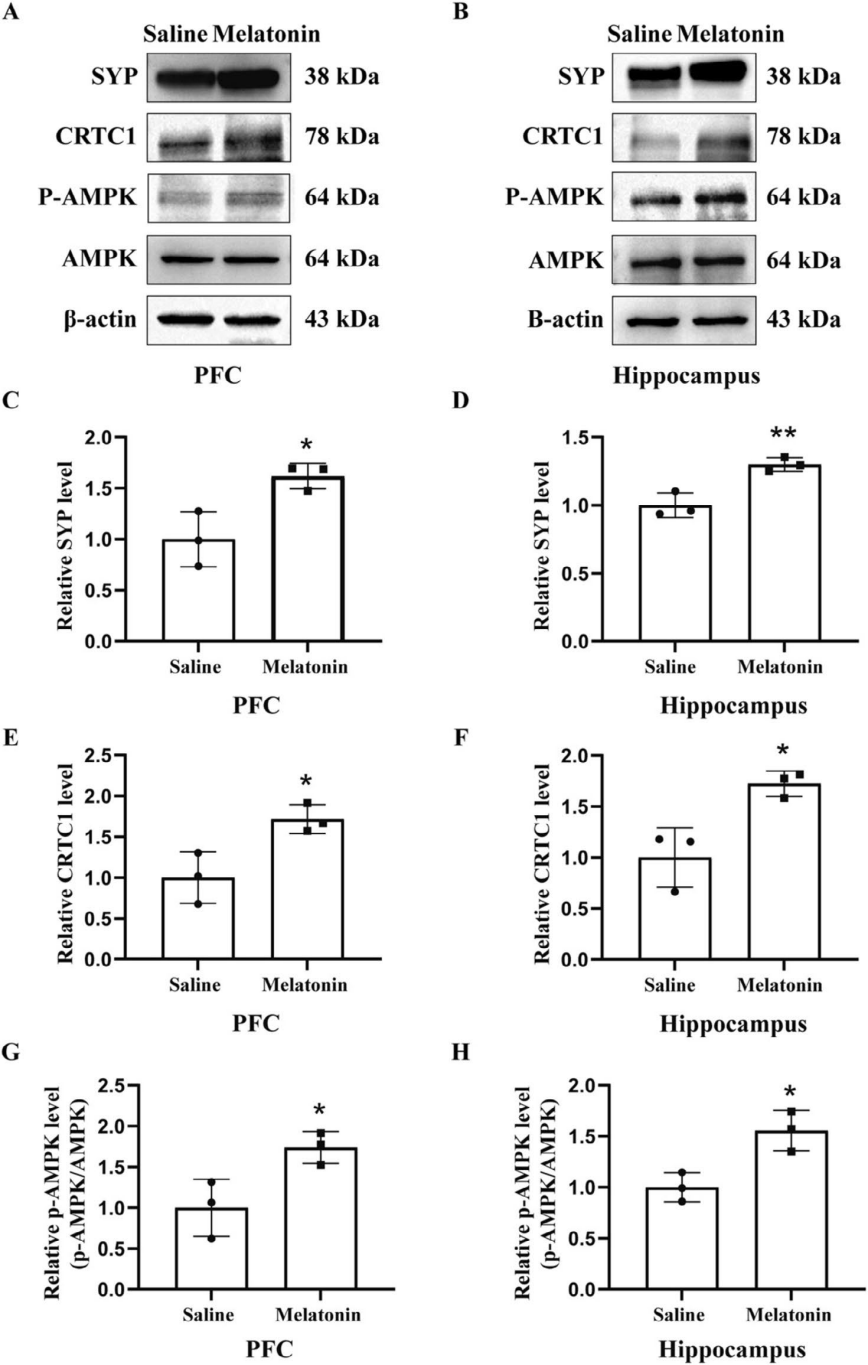
Effect of melatonin treatment on the expression of SYP, CRTC1, and p‐AMPK. Representative immunoblot displayed the bands of SYP, CRTC1, and AMPK in PFC (A) and hippocampus (B) in saline and melatonin‐treated old mice. The relative band intensity of SYP, CRTC1, and AMPK was quantified. Melatonin treatment significantly improved the expression of SYP (C), CRTC1 (E), and p‐AMPK in PFC (G) and SYP (D), CRTC1 (F), and p‐AMPK in the hippocampus (H) *p < 0.05 vs. young. *N* = 3/group. Data were shown as mean ± SEM.

We next examined the effect of melatonin treatment on the expression level of SYP, CRTC1, and p‐AMPK. We demonstrated that 1‐week melatonin treatment significantly reduced aging‐induced decreased expression of SYP (*p* < 0.05, Figure 6A,C for PFC, B,D for hippocampus), CRTC1 (*p* < 0.05, Figure 6A,E for PFC, B,F for hippocampus), and p‐AMPK (*p* < 0.05, Figure 6A,G for PFC, B,H for hippocampus), suggesting that melatonin treatment improves aging‐associated memory loss by regulating the expressions of CRTC1 and AMPK.

### 3.7 Effects of 1 Melatonin Treatment on BBB Integrity and the Protein Expression of Occludin, YAP, and Pdlim5 in the PFC and Hippocampus of Old Mice

**FIGURE 7:**
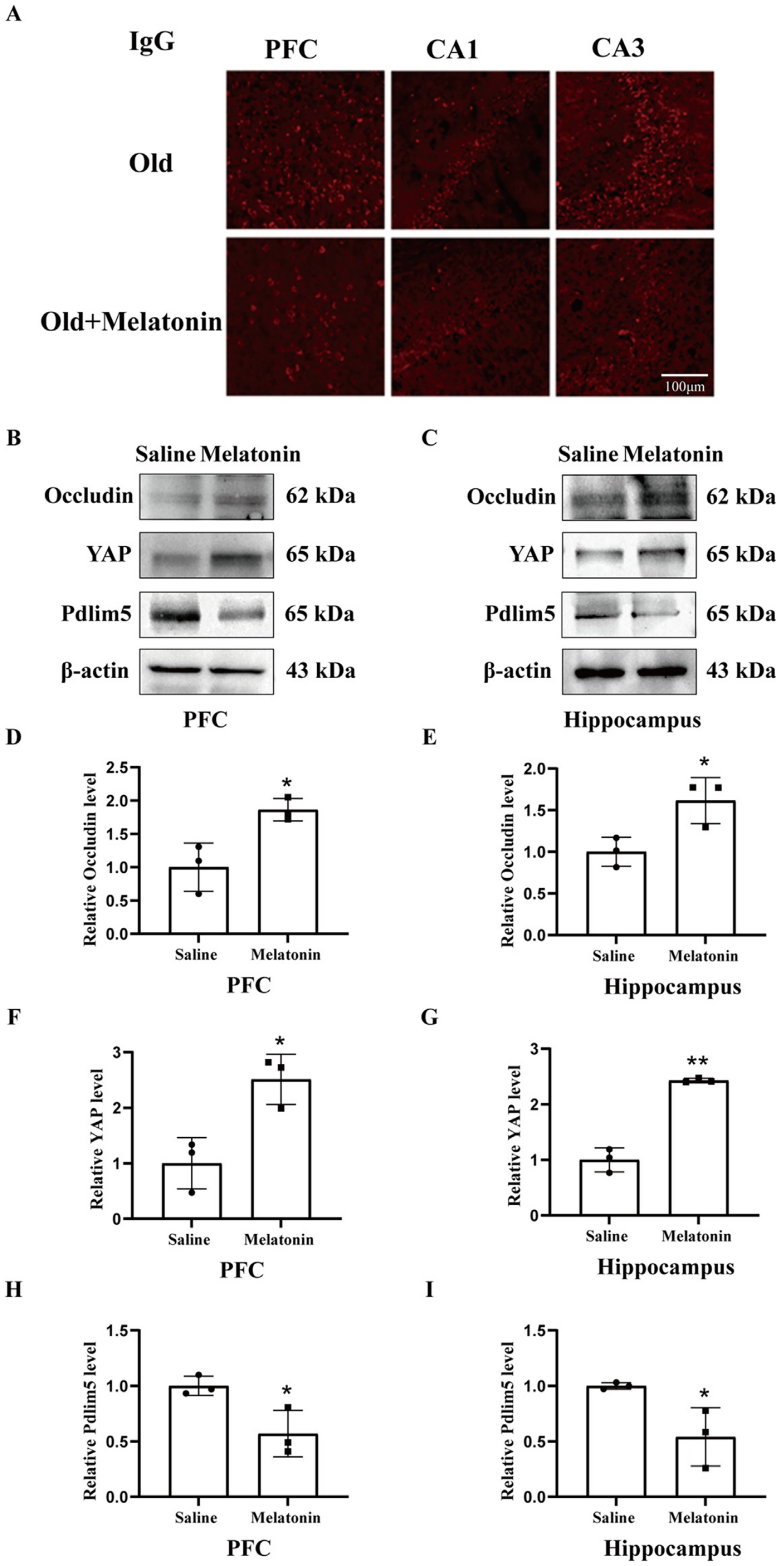
Effect of melatonin administration on the BBB permeability (IgG), expression of occludin and YAP, Pdlim5. Representative fluorescence micrographs demonstrated IgG leakage in the PFC and hippo in saline and melatonin‐treated old mice. Melatonin significantly improved IgG leakage in the PFC and hippocampus. *N* = 3/group. Representative western blot showed the bands of occludin, YAP, and Pdlim5 in PFC (B) and hippocampus (C) in saline and melatonin‐treated old mice. The relative band intensity of occludin, YAP, and Pdlim5 in PFC and hippo was quantified. Melatonin significantly improved the expression of occludin (D), YAP (F), and Pdlim5 (H) in PFC and occludin (E), YAP (G), and Pdlim5 (I) in the hippocampus *p < 0.05. *N* = 3/group. Data were shown as mean ± SEM.

Our data demonstrated that 1 week of melatonin treatment significantly ameliorates aging‐induced BBB damage as reflected by the reduced leakage of IgG (Figure 7A) and occludin degradation in the PFC (*p* < 0.05, Figure 7B,D) and hippocampus (*p* < 0.05, Figure 7C,E). In addition, 1‐week melatonin treatment significantly alleviated aging‐induced YAP downregulation (*p* < 0.05, Figure 7B,F for PFC, C,G for hippocampus) and Pdlim5 upregulation (*p* < 0.05, Figure 7B,H for PFC, C,I for hippocampus), suggesting that melatonin treatment may also improve memory performance in the NOR task through regulating Pdlim5/YAP‐mediated BBB leakage.

## 4 Discussion

**FIGURE 8:**
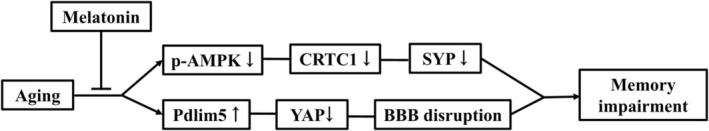
A schema for the molecular mechanism of melatonin's beneficial effect on aging‐produced memory deficit in NOR task in mice. Aging disrupts memory by reducing CRTC1 and AMPK. In addition, the Pdlim5/YAP‐mediated BBB damage in aged mice also plays an important role in aging‐induced memory impairment. Melatonin treatment improved aging‐induced memory damage by increasing CRTC1 and AMPK and maintaining the integrity of BBB.

Previous studies have shown that aging impairs recognition memory in humans and mice [34, 35]. BBB dysfunction is closely related to neurological diseases [36], and decreased melatonin levels have been detected in the brain and blood circulation in elderly people [32]. However, whether there is a link between aging‐induced memory impairment, BBB dysfunction, and low melatonin levels remains unknown. In the present study, we used 12‐month‐old (aged) mice to examine the effect of melatonin on aging‐associated memory decline in the NOR task. Our key findings include (1) aging impaired memory in NOR accompanied by downregulation of SYP, CRTC1, and p‐AMPK in PFC and hippo. (2) Aging‐induced BBB dysfunction is associated with occludin degradation in the PFC and hippo. (3) Pdlim5 is upregulated in the PFC and hippocampus of aged mice, and aging‐related memory impairment is significantly reduced in Pdlim5^−/−^ mice. (4) One‐week melatonin treatment (10 mg/kg) significantly improves aging‐impaired memory in NOR accompanied by an increase in BBB integrity, Pdlim5 downregulation, and upregulation of CRTC1 and p‐AMPK. Taken together, our findings demonstrate that melatonin treatment improves aging‐related memory decline in NOR by downregulating Pdlim5, maintaining BBB integrity, and upregulating CRTC1 and p‐AMPK in aged mice (Figure 8).

It has been reported that aging impairs short‐term [37, 38] and long‐term recognition memory in mice [34, 35]. Notably, age‐induced declines in melatonin levels are functionally important in the aging process [39] and the decreased melatonin levels in humans are proposed to be a predisposing factor for neurodegenerative disorders [40]. For example, decreased melatonin expression has been observed in sporadic Alzheimer disease (sAD) patients, and melatonin can reduce the accumulation of β‐amyloid and improve short‐term memory in streptozotocin‐induced sAD model [41], suggesting that melatonin treatment might be a potential approach to either prevent or slow down the progression of Alzheimer disease [42, 43]. In addition, studies have shown that melatonin alleviates memory deficits in aged rats fed a high‐fat diet by mitigating brain insulin resistance [44]. Moreover, melatonin has been shown to alleviate aging‐induced cognitive deficit by modulating mitochondrial function and cell viability in the PFC and hippocampus [33]. Furthermore, Melatonin treatment may mitigate D‐galactose‐produced memory deficit, neuroinflammation, and neurodegeneration in an aging mice model [18] and improve D‐galactose‐induced aging effects in mice [20]. We demonstrate that melatonin treatment alleviates aging‐induced PFC and hippocampus‐dependent memory loss in the NOR task, further supporting that melatonin supplementation is a potential strategy to ameliorate aging‐associated memory loss.

In this study, our results indicate that aging significantly downregulates the expression of CRTC1 in the PFC and hippocampus, whereas melatonin treatment alleviates these changes and is accompanied by memory enhancement. CRTC1 has emerged as a critical regulator of gene expression targeting longevity promotion, and dysregulation of CRTCs is associated with aging [5]. CRTC1 is important in forming fear memory [45, 46], stroke‐induced memory loss [47], schizophrenia [23], and LPS [48]‐associated working memory deficit. In addition, dysregulated CRTC1 in the hippocampus is known to contribute to Aβ oligomer‐induced memory impairment [49], and CRTC1 function is disrupted during memory formation in neurodegeneration [50], suggesting that CRTC1 can be regarded as a potential target to ameliorate aging‐generated episodic memory impairment.

In this study, we found that aging reduces the levels of p‐AMPK in the PFC and hippo, and melatonin treatment improves memory by ameliorating this change, suggesting that the reduction of p‐AMPK in the PFC and hippo is associated with aging‐induced memory deficit in the NOR task. In addition, AICAR, an AMPK agonist, has been shown to improve memory in young and old mice [51] and metformin treatment has been shown to prevent Aβ accumulation and memory loss in APP/PS1 mice [52], suggesting that activating AMPK can be a potential way to reduce aging‐associated memory deficit.

Aging is known to impair both the structural integrity and functional capabilities of the BBB^6^. Our results in this study indicated that aging significantly disrupts BBB integrity in the PFC and hippocampus, and melatonin treatment alleviates aging‐induced BBB damage accompanied by memory enhancement. BBB disruption may be a primary factor in the onset of aging‐related neurodegenerative diseases, rather than solely a consequence of them [53]. For example, BBB damage, particularly within the hippocampus, is an early hallmark of aging in the human brain and may play a crucial role in the development of memory deficits [28]., and BBB damage is an early event that contributes to subsequent memory impairment and neurodegeneration in a diabetic, insulin‐resistant mouse model [54]. In addition, BBB impairment and collagen aggregation in perivascular regions precede microvessels sparse and memory deficit in an animal model of chronic hypertension [55]. Therefore, maintaining the integrity of the BBB can be a hopeful strategy to prevent aging‐generated memory impairment.

Endogenous IgG leakage and exogenously supplied Evan's blue dye tests are widely used to determine the integrity of the BBB [56, 57]. There are reports that Evan's blue leakage is only at the symptomatic stage. IgG leakage appears to be a highly sensitive indicator of BBB disruption, often appearing early in the disease process [58]. Given its sensitivity and early detection capabilities, we selected IgG leakage as a marker for BBB damage in this study.

In this study, we further show that aging‐induced impairment of NOR memory is accompanied by the Pdlim5 upregulation and the memory impairment can be alleviated in Pdlim5^−/−^ mice. Pdlim5 upregulation is a key contributory factor in BBB damage after stroke and knockout Pdlim5 could alleviate this damage [14]. In addition, Pdlim5/YAP is shown to be critically involved in BBB damage following stroke [15]. Therefore, in aging‐induced memory impairment, Pldim5 may modulate the BBB by regulating YAP. In this study, we showed that aging is accompanied by YAP downregulation and melatonin treatment can upregulate YAP expression. Furthermore, the miR‐17 ~ 92 cluster is crucial for cognition and can modulate neurogenesis in the hippocampus by regulating Pdlim5 in neural stem cells [59], suggesting that aging may affect Pdlim5 through regulating miR‐17 ~ 92.

### 4.1 Limitation

This study exclusively utilized male mice. Moreover, as testing was conducted at a single time point, it remains unclear whether the observed memory impairments are transient or enduring. Within the brain, melatonin undergoes metabolic conversion to N1‐acetyl‐N2‐formyl‐5‐methoxykynuramine (AFMK) and subsequently to N1‐acetyl‐5‐methoxykynuramine (AMK). Notably, AMK has been demonstrated to significantly enhance long‐term object memory. It is well established that hippocampal AMK levels decline with age [60], highlighting its potential as a prophylactic intervention for dementia [61]. Previous research has shown that both melatonin and AMK administration can improve long‐term memory in various mouse strains, including melatonin‐deficient ICR mice [19] and melatonin‐producing C3H/He mice [62], as assessed by the Novel Object Recognition (NOR) test. Although melatonin primarily exerts its effects through its receptors MT1 and MT2, the specific receptor subtype mediating the observed effects of melatonin in this study remains undetermined. Future studies are necessary using the MT1/MT2 antagonist luzindole to confirm to what extent the observed effects occur via the two receptors and using 4P‐PDOT to investigate whether these effects are mediated by MT1, MT2, or both.

In summary, our findings suggest that aging impairs memory in NOR by downregulating the expression of SYP, CRTC1, AMPK, and Pdlim5‐mediated BBB damage, and melatonin treatment improves memory by upregulating CRTC1 and AMPK, as well as maintaining the integrity of BBB by downregulating Pdlim5.

## Author Contributions

Yanping Wang, Conceptualization, Writing – original draft, Data analysis, Funding acquisition. Xinyu Zhang: Experimental operation, and Data analysis. Hui Guo, Experimental operation and Data analysis. Shuxia Qian: Writing – original draft and figure preparation. Hailun Fang, Data analysis. Xiaoqiang Wu: Data analysis. Yufei Shen: Writing – original draft. Congying Xu: Data analysis. Beiqun Zhou: Data analysis. Chun Guo, Writing – review and editing. Xudong Lu: Writing – review and editing. Xiaoling Zhang: Writing – review and editing. Xinchun Jin: Writing – review and editing and funding acquisition. All authors have read the paper and agreed with the submission.

## Conflicts of Interest

The authors declare no conflicts of interest.

## Data Availability

The data that support the findings of this study are available from the corresponding author upon reasonable request.
